# Linoleate-enriched diet increases both linoleic acid esterified to omega hydroxy very long chain fatty acids and free ceramides of canine stratum corneum without effect on protein-bound ceramides and skin barrier function

**DOI:** 10.1007/s00403-018-1845-5

**Published:** 2018-07-11

**Authors:** Iuliana Popa, Adrian L. Watson, Audrey Solgadi, Christina Butowski, David Allaway, Jacques Portoukalian

**Affiliations:** 1 Faculty of Pharmacy, University Paris-Sud, Chatenay-Malabry, France; 2 Royal Canin SAS, Aimargues, France; 3 UMS 3679 CNRS, Faculty of Pharmacy, University Paris-Sud, Chatenay-Malabry, France; 4 Waltham Centre for Pet Nutrition, Leics, UK; 5 LBTM1 “Fundamental, clinical and therapeutic aspects of the skin barrier function”, University of Lyon-1, Lyon, France

**Keywords:** Acylacids, Ceramides, Diet, Dogs, Linoleic acid, Stratum corneum

## Abstract

Few studies have investigated the influence of increased amounts of dietary linoleic acid on the epidermal lipid biochemistry and TEWL in healthy subject. The influence of dietary linoleic acid on canine stratum corneum (SC) lipids was studied by feeding two groups of five dogs differential amounts of linoleic acid (LA) for three months. SC was harvested by tape stripping and lipids were analyzed by thin-layer chromatography and mass spectrometry. The dogs that were fed the higher concentration of LA showed high increases in the contents of both linoleic acid and free ceramides in the SC, whereas the protein-bound ceramide content was unchanged. Acylacids that represent the esterified form of linoleic acid in omega hydroxy very long chain fatty acids (ω-OH VLCFA) accounted for most of the elevation of LA, whereas the concentration of the free form was not significantly changed. Corroborating the absence of change in the protein-bound ceramides content of healthy dogs SC, TEWL was nearly unaffected by the linoleic acid-enriched diet.

## Introduction

**Fig. 1:**
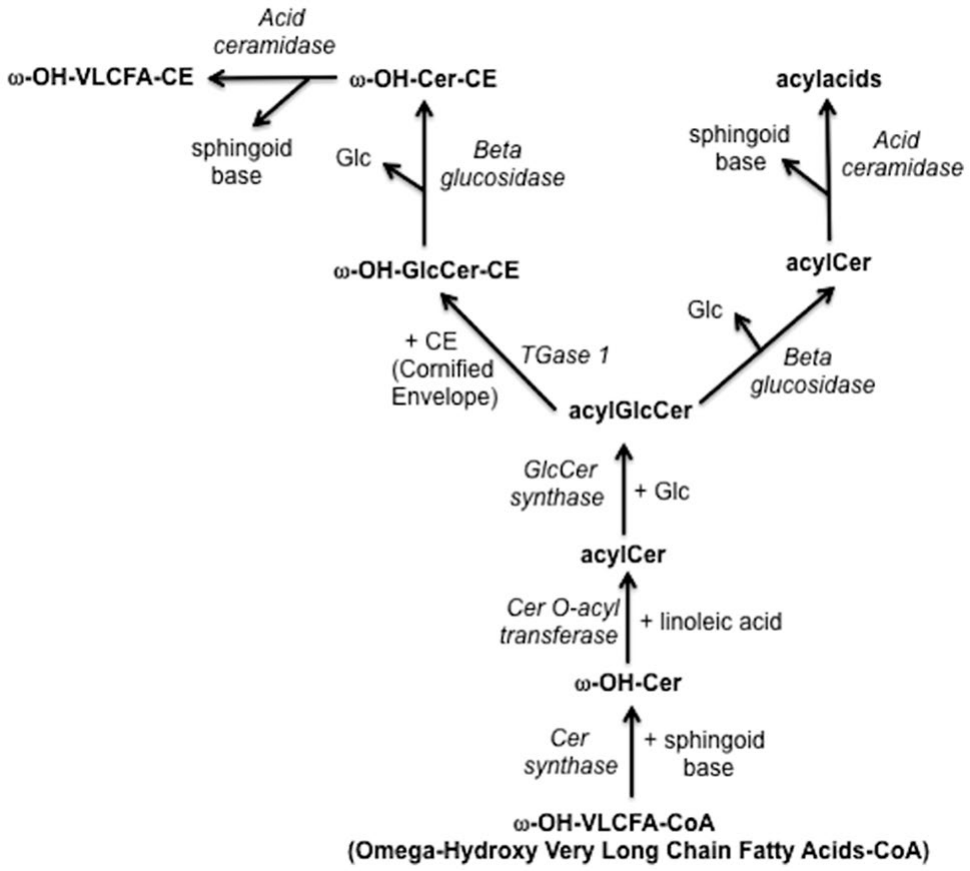
Pathway of lipid biosynthesis in epidermis leading to acyl acids containing linoleic acid. The synthetized lipids are: ω-OH-VLCFA-CoA (Omega-Hydroxy Very Long Chain Fatty Acids-CoA), ω-OH-Cer (Omega-Hydroxy-Ceramide), acylCer (acyl-Ceramide), acylGlcCer (acyl glucosyl-ceramide), ω-OH-GlcCer-CE (Omega-Hydroxy acyl glucosyl-ceramide –Cornified Envelope), ω-OH-Cer-CE (Omega-Hydroxy ceramide-Cornified Envelope), ω-OH-VLCFA-CE (omega-hydroxy very long chain fatty acids-cornified envelope). Substrate lipids are: Glc (Glucose), sphingoid base, linoleic acid. The involved enzymes are: Cer synthase (ceramide synthase), Cer O-acyl transferase (ceramide O-acyl transferase), GlcCer synthase (Glucosyl synthase), TGase 1 (Transglutaminase 1), Beta glucosidase, acid ceramidase

Linoleic acid C18:2 is a polyunsaturated fatty acid which is only synthesized by plants. For humans and other mammals it is, therefore, an essential dietary requirement. A deficient diet results in, amongst other things, the development of a scaly skin condition with concomitant increase in transepidermal water loss (TEWL) [6], a condition known as Essential Fatty Acid Deficiency. In the epidermis, LA is incorporated into an esterified form on omega-hydroxy very long chain fatty acids (ω-OH VLCFA) with 30–34 carbon atoms. The pathway of biosynthesis in the epidermis leading to the presence of linoleic acid-containg acylacids is shown in Fig. 1. These fatty acids are mainly found in the form of acylceramides [32] and acylacids [3], both of which are critical components for maintaining the integrity of the epidermal water permeability. The coupling of LA to ω-OH VLCFA seems a critical point; topical application of free LA has no effect on TEWL, whereas LA ester-bound to ω-OH VLCFA results in reduced TEWL in essential fatty acid-deficient rats [14] which have previously been fed oleic acid instead of linoleic acid, thereby inducing disruption of acylglucosylceramides and acylceramides [18]. It has also been reported that LA can modulate lipid metabolism by activating peroxisome-proliferator-activated receptors alpha (PPARSα) [8].

Few studies have been conducted on the effect of dietary linoleic acid on skin lipid biochemistry in healthy subjects. Most investigations have focused on oral intake of linoleic acid in essential fatty acid deficiencies. One report did show a beneficial effect of LA by reducing senile dryness in healthy middle-aged American women [5]. The present study, using healthy adult dogs, was designed to investigate the influence of dietary linoleic acid significantly above the normal recommended daily requirement on the lipids of canine SC and skin barrier function as measured by TEWL. The selection of the two linoleic acid concentrations tested in this study (4 g/Mcal and 10 g/Mcal) was based on the range observed in typical pet foods on the market. Many grocery products contain linoleic acid around the 4–5 g/Mcal range, whereas higher quality feeds typical have linoleic acid spanning 7–10 g/Mcal [1]. The 10 g/Mcal concentration, therefore, lies at the top end of the premium type commercial diets.

## Methods

### Ethical statement

Dogs on this study were housed in purpose-built, environmentally enriched housing at the Waltham Centre for Pet Nutrition (Waltham, U.K.). All housing, care and procedures were in keeping with the requirements of the Animals (Scientific Procedures) Act 1986 and the study approved by Waltham’s Animal Welfare and Ethical Review Body.

### Diets and sampling

All dogs were fed nutritionally balanced and complete diets for the duration of the study according to individual metabolic energy requirements (bMER 95 kcal × BW^0.75^) [19]. The linoleic acid concentration of the two diets was adjusted relative to the oleic acid component via changes to the contribution of fats derived from coconut, soya or pork. The analyses of the principle components of the two diets are shown in Table 1. Increased LA in the supplemented diet was largely compensated by a reduction in palmitate and oleate. At the start of the study, ten Labrador Retrievers were fed a diet containing 4 g/Mcal linoleic acid for twelve weeks to establish baseline conditions. Subsequently, the dogs were assigned to two groups of 5, matched for age and sex. One group, Group 1, continued on the baseline diet and the other, Group 2, changed to a similar diet adjusted to 10 g/Mcal linoleic acid for a further 12 weeks. Immediately prior to the diet switch, and then following 8 and 12 weeks, a series of 10 tape strip samples of SC were sequentially taken from the same abdominal area of epidermis. Strips were placed into clean plastic tubes and immediately stored at − 80 °C until processing.

**Table 1:** Dietary analysis of baseline and supplemented diets

Diet composition	Baseline	Supplemented
Ash % (w/w)	6.2	6.5
Cellulose % (w/w)	1.8	1.6
Fat % (w/w)	13.3	13.2
Protein % (w/w)	25.4	24.2
Linoleic acid (g/Mcal)	4.05	10.07
Arachidonic acid (g/Mcal)	0.129	0.163
EPA/DHA (g/Mcal)	0.15	0.15

### Transepidermal water loss (TEWL)

TEWL measurements were taken at the end of the pre-feed period, and subsequently following 4, 8 and 12 weeks of the differential feeding period. Five separate measurements were taken at each time point for each dog. The dogs were assessed in a quiet, draft-free room with consistent environmental conditions. Measurements were taken using a Cutometer MPA580 fitted with a Tewameter Triple TM 330 T (3 Probes measure TEWL simultaneously) placed 1–2 inches to one side of the lumbar spine, first parting the hair coat so as to ensure good contact of the probe with the skin. The lumbar region of the animal provided a good surface for orientation of the 3 probes. Prior to the start of the study, the dogs were conditioned to remain stationary for 1 min at a time to improve stability of readings.

### Lipid extraction and analysis

Tapes were placed in a glass tube containing hexane–isopropanol 5:1 (v/v) to remove the adhesive without solubilizing the cellular lipids. After sonication on ice for 5 mn, the tapes were removed and the tubes were centrifuged at 1500×*g* to recover the corneocytes. The corneocyte pellets were extracted at room temperature in chloroform–methanol 2:1 (v/v), centrifuged then extracted twice more with methanol only [22]. The pooled supernatants were evaporated to dryness under nitrogen and the lipid residues were taken up with diethylether. The protein-bound lipids were extracted from the cell pellets residues by mild saponification with KOH 0.1 N in methanol–water 10:1 (v/v) for 2 h at 50 °C. After neutralization, the protein residue was removed by centrifugation and assayed by the Coomassie Blue method.

Free and protein-bound lipids extracted from the strips were fractionated sequentially into several lipid classes on LC-NH2 silica gel cartridges (Supelco, L’Isle d’Abeau, France) as previously described [22]. Briefly, the fractions containing neutral lipids, free fatty acids, and ceramides (glucosylceramides and phospholipids were absent) were eluted respectively with chloroform, chloroform–methanol 23:1 (v/v) and diisopropylether-acetic acid 98:3 (v/v). The fractions were analyzed by thin-layer chromatography, along with known amounts of standards. The plates were visualized with a spray reagent (3% copper acetate in 8% phosphorous acid) by heating in an oven at 150 °C. For each plate, the quantification was performed three times by scanning densitometry with a CS-930 Chromatoscan (Shimadzu, Kyoto, Japan) and the variations were always below 5%.

### Assay of linoleic acid by LC-APCI/MS

The fatty acid fractions extracted from SC tape strips and purified by chromatography on the LC-NH2 cartridges were taken up in chloroform and analyzed by liquid chromatography-mass spectrometry (LC-MS) without derivatization. To quantify the amount of linoleic acid, 2 µg of standard pentadecanoic acid C15:0 (Sigma, L’Isle d’Abeau, France) that represents less that one percent of free fatty acids in SC [20] was added to each fraction. A 5 µl sample was injected in a RSLC Dionex-U300 column coupled to a LTQ-Orbitrap Velos Pro mass spectrometer (Thermofisher Scientific, Villebon-sur-Yvette, France) equipped with an atmospheric-pressure chemical ionization (APCI) interface. The mass spectra were recorded with high resolution (100,000) from *m*/*z* 200 to *m*/*z* 800. The amount of linoleic acid in each fatty acid sample was calculated from the intensity of the ion *m*/*z* 279, knowing that the intensity of the standard C15:0 ion *m*/*z* 241 corresponded to 2 µg of pentadecanoic acid exogenously added to each sample.

### Statistics

For TEWL data a mixed effects model was used where TEWL was modelled against Week, Diet and the Week by Diet interaction as fixed effects with a random effects structure of Probe nested in Repeat nested in Week nested in Dog. Planned contrasts were applied comparing the ‘change from week 0’ between diets and testing for between week differences within each diet. Significance at *P* < 0.05. For lipid analysis, the quantitative results obtained by scanning densitometry were compared using Student’s *t* test, *P* = 0.05 being accepted as significant.

## Results

**Fig. 2:**
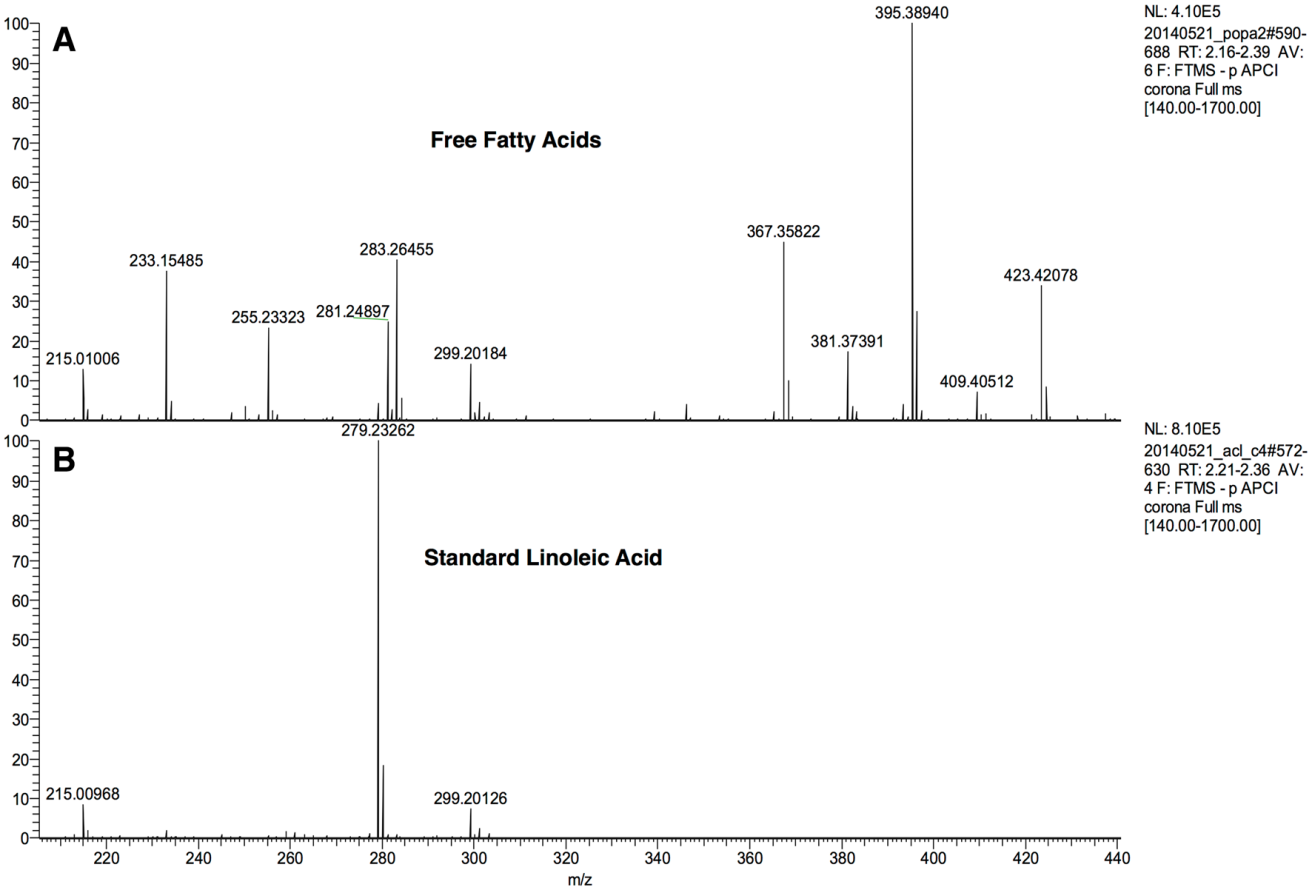
Identification of linoleic acid in the free fatty acid fraction purified from canine stratum corneum by APCI LC-MS. RSLC Dionex-U3000 coupled to LTQ-Orbitrap Velos Pro, Thermofisher Scientific with an APCI source. **a** Free fatty acids spectra. **b** Linoleic acid spectra- standard use

As mentioned in the previous section, the two groups of dogs followed a specific linoleic-enriched diet. Tape strip lipid samples taken from the SC of each dog were assessed for free linoleic acid at *m*/*z* 279 (Fig. 2), along with the ions of the major fatty acids from C14 to C30 in the *m*/*z* 240–460 range.

**Fig. 3:**
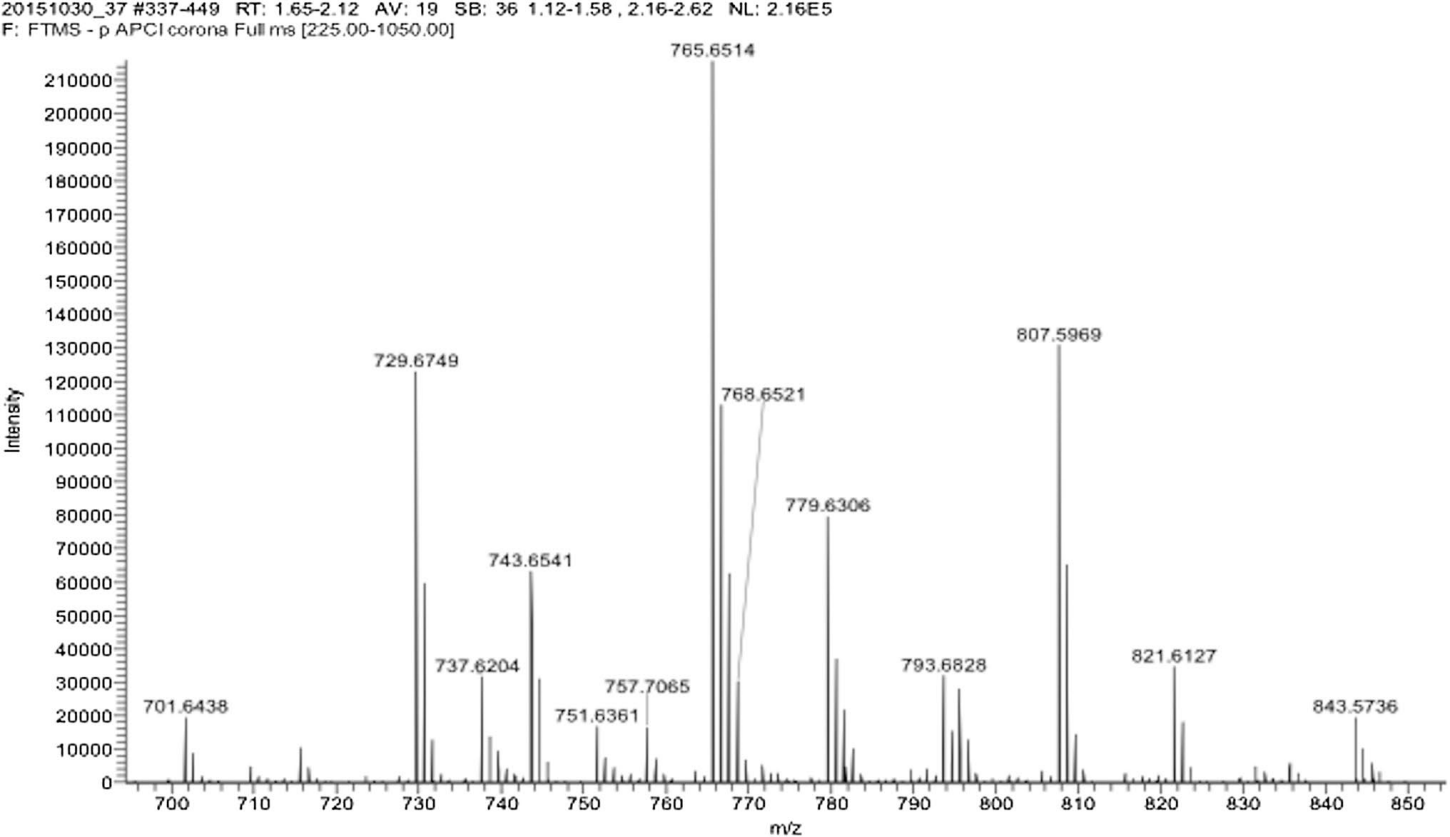
Presence of linoleic acid in its free form and its acylacid forms in the free fatty acids (FFA) fraction shown by APCI LC-MS

**Fig. 4:**
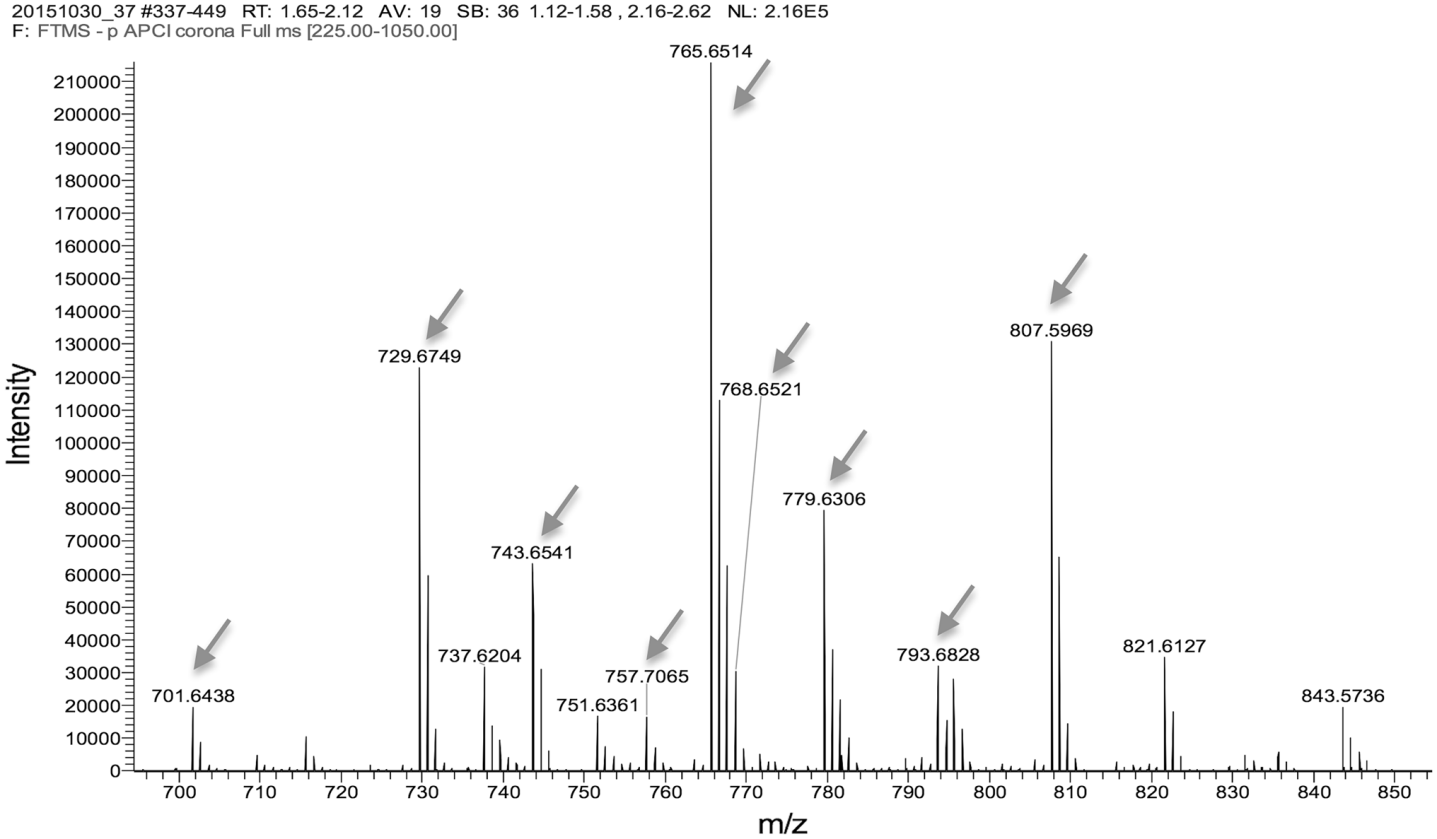
MS spectrum of acylacids (*m*/*z* 700–900). (Arrows corresponding to the data in Table 2.)

**Fig. 5:**
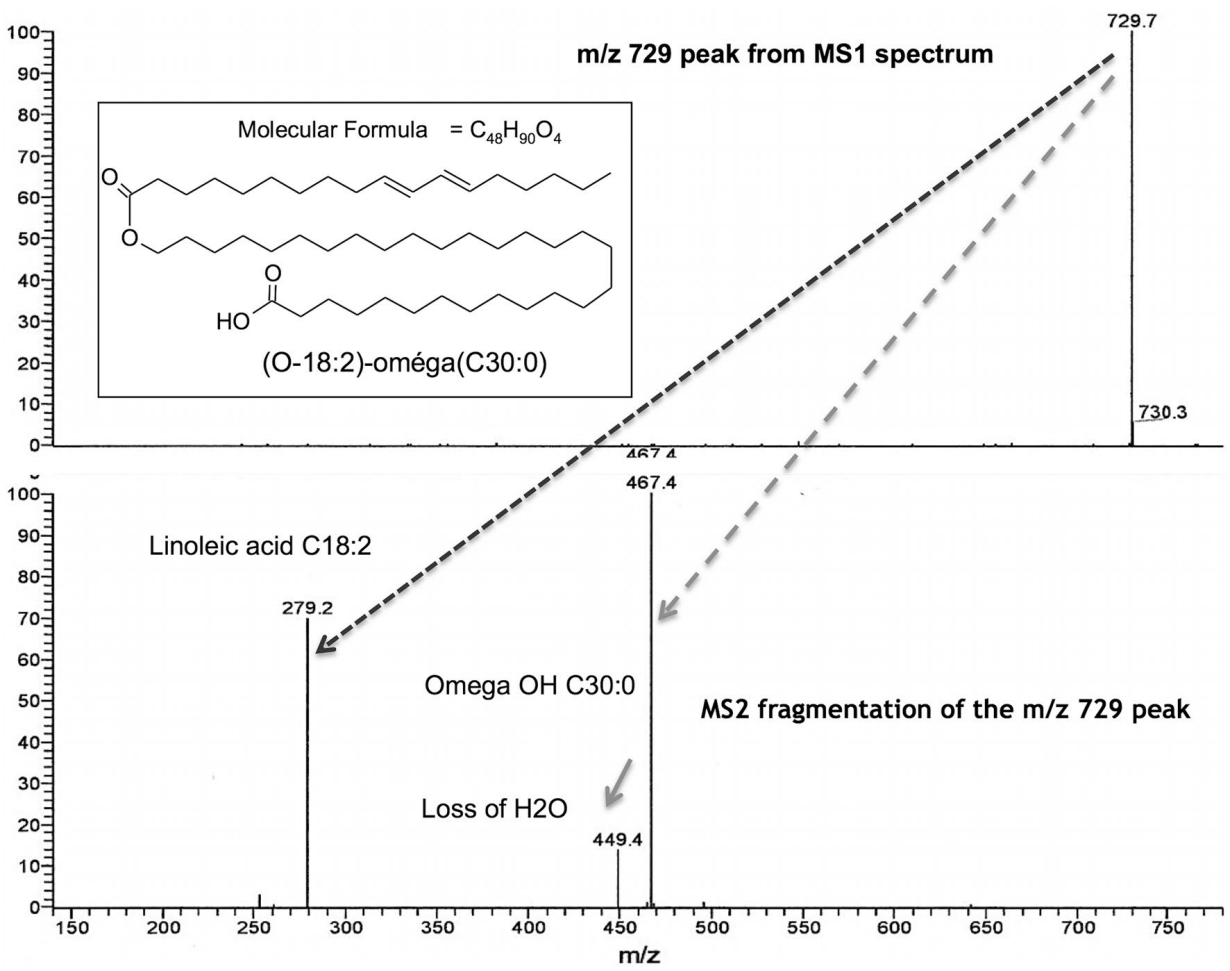
**a** Chemical structure of the omega C30:0; **b** MS spectrum of acylacid moiety (*m*/*z* peak at 729) and its MS2 spectrum CID fragmentation into linoleic acid and omega OH C30:0 in negative mode

**Fig. 6:**
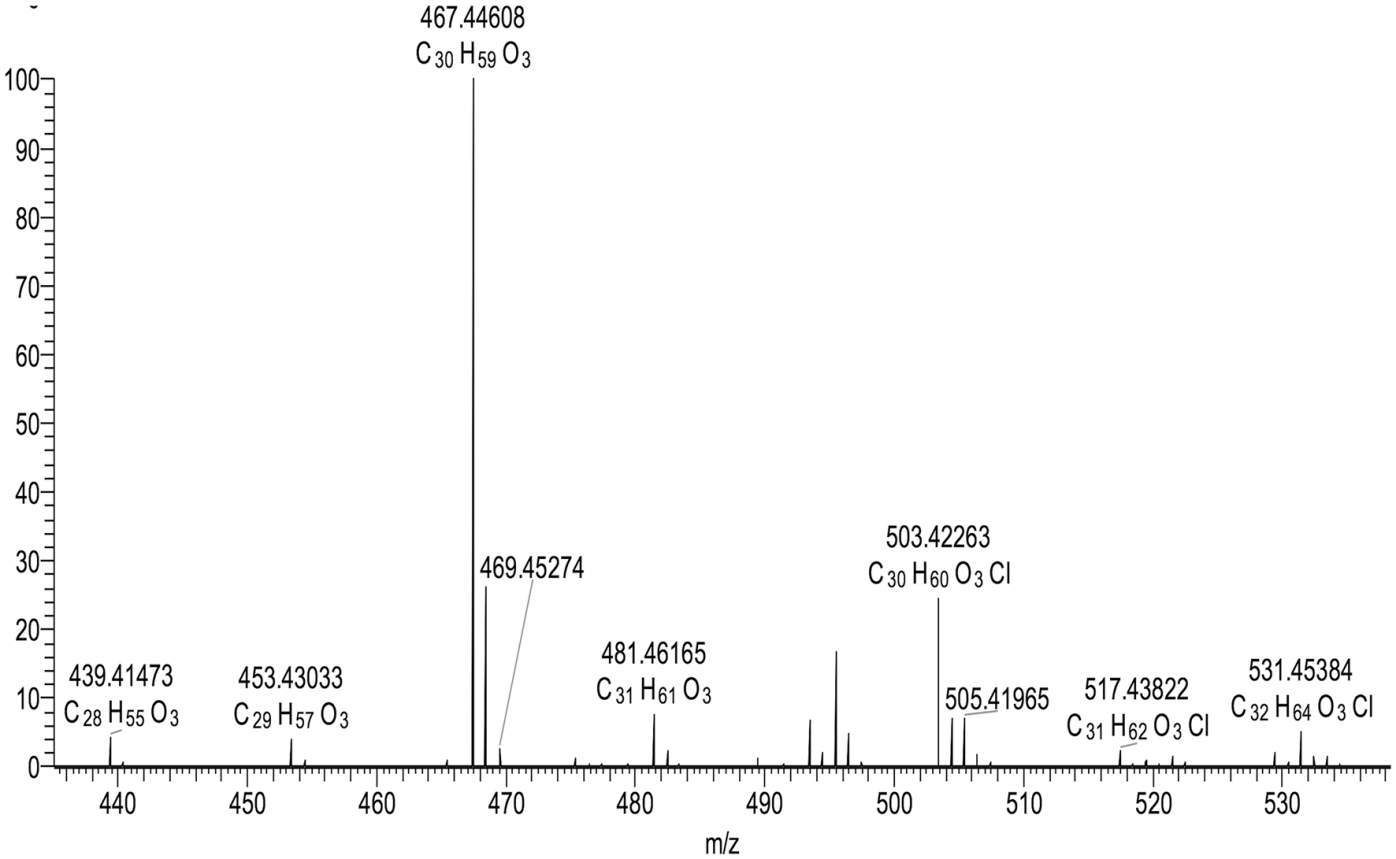
Presence of omega hydroxy very long chain fatty acids (ω-OH VLCFA) (m/z 415–570) in the free fatty acid (FFA) fraction

In a broader range from *m*/*z* 240 to 800 (Fig. 3), as well as the free fatty acids of SC clearly separated in the *m*/*z* 240–460 range, there was a second group of fatty acids in the *m*/*z* 700–800 corresponding to acylacids that are esters of ω-OH VLCFA [3]. An extended spectrum of acyl acids is shown in Fig. 4. Using MS/MS (MS2), the acylacid ions were cleaved into linoleic acid C18:2 and ω-OH VLCFA of different lengths and degrees of unsaturation. Figure 5 shows that, after MS2 in the negative ion mode, the compound at *m*/*z* 729 released C18:2 (*m*/*z* 279) and ω-OH C30:0 (*m*/*z* 467). The results of MS2 experiments on the major ions recorded in the range of *m*/*z* 700–800 shown in Fig. 4 are listed in Table 2. As the lipids were solubilized in chloroform, adducts with Cl^−^ were formed for each anion. The MS2 ionization generally induced a loss of minus 18 for each fragment, corresponding to one molecule of water. The fragments released upon MS2 from the ions at *m*/*z* 757 and 793 (757 + Cl^−^) were in nearly equal proportions (*m*/*z* 279 and *m*/*z* 281, not shown), and were assigned respectively to linoleic acid C18:2 and oleic acid C18:1. The proportion of oleic acid was found to be about tenfold lower than that of linoleic acid in acylacids, and there were no detectable amounts of palmitic and stearic acids, both of which have previously been reported in the acylacid fraction of human epidermis by Bowser et al. [3], using gas chromatography on methyl ester derivatives. Our data show that almost all the acylacid ions released linoleic acid, along with ω-OH VLCFA that were also present in small amounts in the free fatty acid fractions of canine SC (Fig. 6).

**Table 2:** Results of the MS2 fragmentation of the acylacids recorded from m/z 200 to m/z 900

M − H	M + Cl	Formula	Fragments	Structures
701.643	737.620	C46H85O4	439 ; 421 ; *279*	O–C18:2-ωOH C28:0
729.674	765.651	C48H89O4	467 ; 449 ; *279*	O–C18:2-ωOH C30:0
743.666	779.666	C49H92O4Cl	481 ; 463 ; *279*	O–C18:2-ωOH C31:0
757.706	793.682	C50H93O4	495/493 ; 475/477 ; *279*/*281*	O–C18:2-ωOH C32:0 and O–C18:1-ωOH C32:1
783.700	819.700	C52H96O4Cl	521 ; 503 ; *279*	(O–C18:2)-ωOH C34:0

The corresponding fragmentations, formulas and the molecular weights (M − H and M + Cl) are presented after APCI-LC–MS. The major acylacid fragments (showed in Fig. 3) are at *m*/*z* 467 [omega hydroxy C30:0 (O–C18:2-ωOH C30:0)], *m*/*z* 481 [omega hydroxy C31:0 (O–C18:2-ωOH C31:0)], *m*/*z* 495 [omega hydroxy C32:0 (O–C18:2-ωOH C32:0)], and *m*/*z* 503 [omega hydroxy C34:0 (O–C18:2)-ωOH C34:0]. The major free acids fragments are *m*/*z* 279 for linoleic acid (C18:2) and m/z 281 for oleic acid (C18:1)

**Fig. 7:**
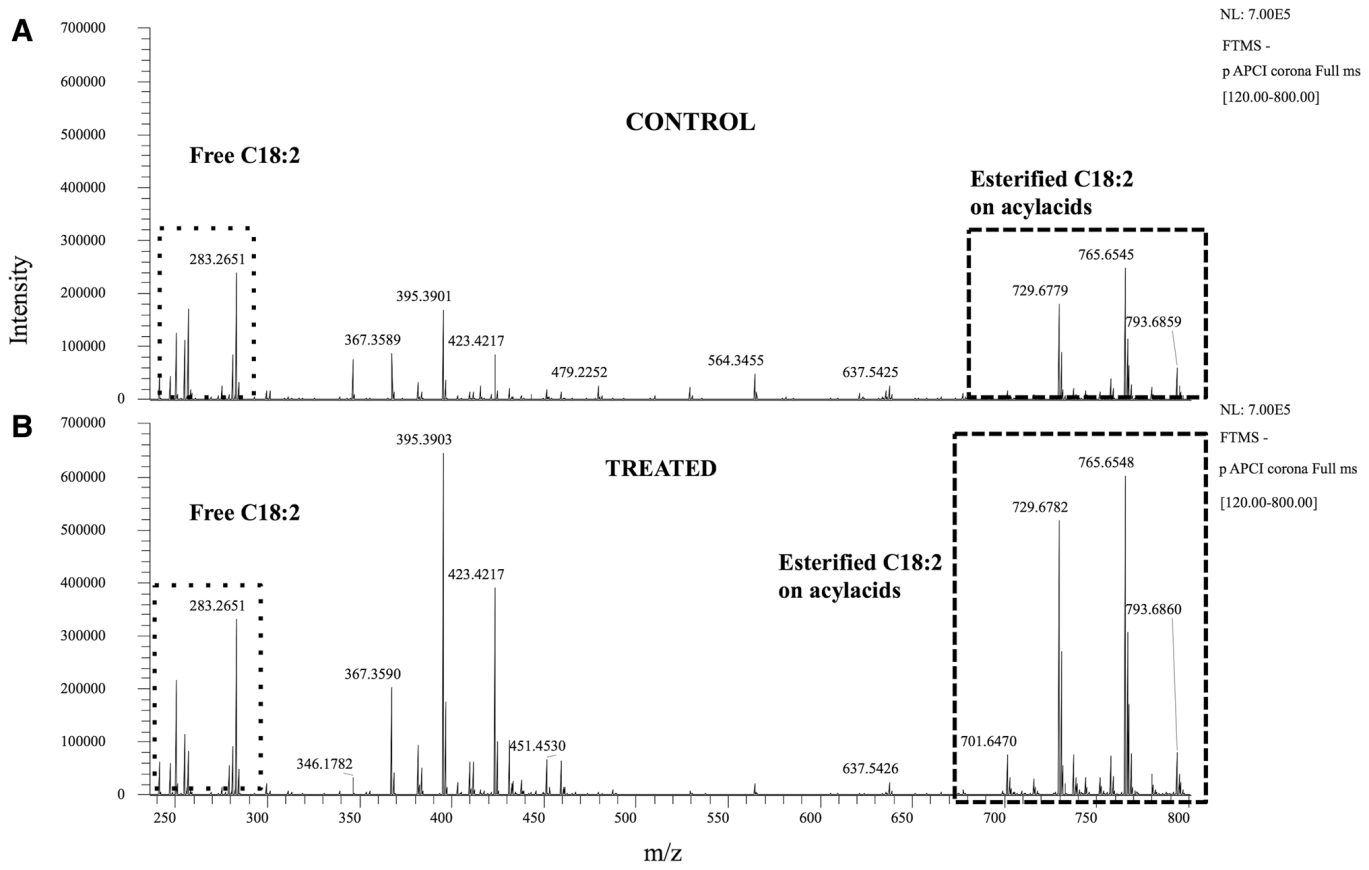
MS spectrum of the fatty acid fraction of the Stratum Corneum of a dog from group 2. A: 3 months control; B: post 6 months treatment

The concentrations of C18:2 in the SC of dogs fed for three months with the higher linoleic acid diet are given in Table 3. The analysis of protein-bound fatty acids showed that the linoleic acid concentration was negligible in this fraction. Moreover, these lipids are released upon saponification and it is well known that alkaline conditions result in the loss of information obtained by mass spectrometry regarding the esterified intact compounds, making it impossible to reconstruct their structures from the hydrolyzed fragments [4]. We confirmed this fact by observing that treating samples of free fatty acids with alkaline conditions did not increase the m/z 279 obtained by mass spectrometry, despite the fact that all of the acylacids were hydrolyzed, as illustrated by the disappearance of the corresponding peaks (not shown). The concentration of linoleic acid in the SC of the group 1 dogs (fed daily with 4 g/Mcal for 6 months) did not change significantly between 3 and 6 months of feeding, whereas that of group 2 dogs (fed daily with 4 g/Mcal LA for 3 months, followed by 10 g/Mcal LA for the next 3 months) increased significantly between 3 and 6 months of supplementation (*p* < 0.05 by Student *t* test). As shown in Table 3, the augmentation of linoleic acid in the dogs of group 2 did not modify the LA distribution between the free and esterified forms; this remained the same with only about 10% of linoleic acid present as the free fatty acid. Figure 7 illustrates the increase in free and ester-bound linoleic acid in one dog of group 2.

**Table 3:** Linoleic acid in the free fatty acid (FFA) fractions of Stratum Corneum samples from the 2 groups of dogs

Feeding group	Dog number		C18:2 in free lipids of 10 strips					
			Prefed 12 weeks			After 24 weeks		
			Total (µg/mg protein)	% as free C18:2	% as acylacid	Total (µg/mg protein)	% as free C18:2	% as acylacid
	1		32.5	11.4	88.6	31.4	12.1	87.9
Group 1	2		14.6	10.7	89.3	23.4	9.6	90.4
	3		43.9	7.9	92.1	39.5	11.5	88.5
	4		23.1	9.8	91.2	30.2	10.7	89.3
	5		14.4	10.3	89.7	21.4	11.2	88.8
mean ± SD (*n* = 5)			25.7 ± 13.0			29.2 ± 6.8		
		6	55.4	9.9	90.1	95.9	12.4	87.6
Group 2		7	11.8	8.6	91.4	100.4	10.5	89.5
		8	54.0	11.5	88.5	59.5	10.1	89.9
		9	19.1	10.9	89.1	27.3	8.7	91.3
		10	49.6	10.4	89.6	79.4	11.1	88.9
mean ± SD (*n* = 5)			37.9 ± 18.7			72.5 ± 26.8*		

Values for C18:2 as free and acylacid forms are obtained after lipid purification of 10 strips taken from the same dog after respectively 12 and 24 weeks feeding

*Significantly different from the prefed value of group 2 (*p* > 0.05)

The lipids of canine SC are made of free cholesterol, fatty acids and ceramides [23]. As well as increasing the overall linoleic acid content of SC, feeding the dogs with the linoleic acid-enriched diet also induced a significant increase (about 60%) in the free ceramide content (Table 4). Conversely, the protein-bound ceramide content was not significantly modified. There was also no change in the free cholesterol content, as assessed by high-performance thin-layer chromatography (TLC) (not shown). Scanning densitometry of the ceramide TLC plates showed that the increase was evenly distributed among all ceramide species (not shown). There was no significant change of ceramide content in the SC of group 1 dogs fed the low linoleic acid diet (not shown).

**Table 4:** Increase of ceramides in stratum corneum of group 2 dogs fed 24 weeks with LA-enriched diet

	Group 2 dogs prefed 12 weeks	Group 2 dogs fed 24 weeks
Free ceramides (µg/mg proteins ± SD)	132.6 ± 20.4, *n* = 5	212.2 ± 23.5, *n* = 5*
Protein-bound ceramides (µg/mg proteins ± SD)	33 ± 10.8, *n* = 5	38 ± 9.9, *n* = 5

Average values were obtained after scan densitometry of five ceramides HPTLC plates processed from the strips of five dogs from group 2. The ceramide quantities were normalized against the protein content of the strips

*Significantly different from the prefeed control (*p* < 0.05)

**Fig. 8:**
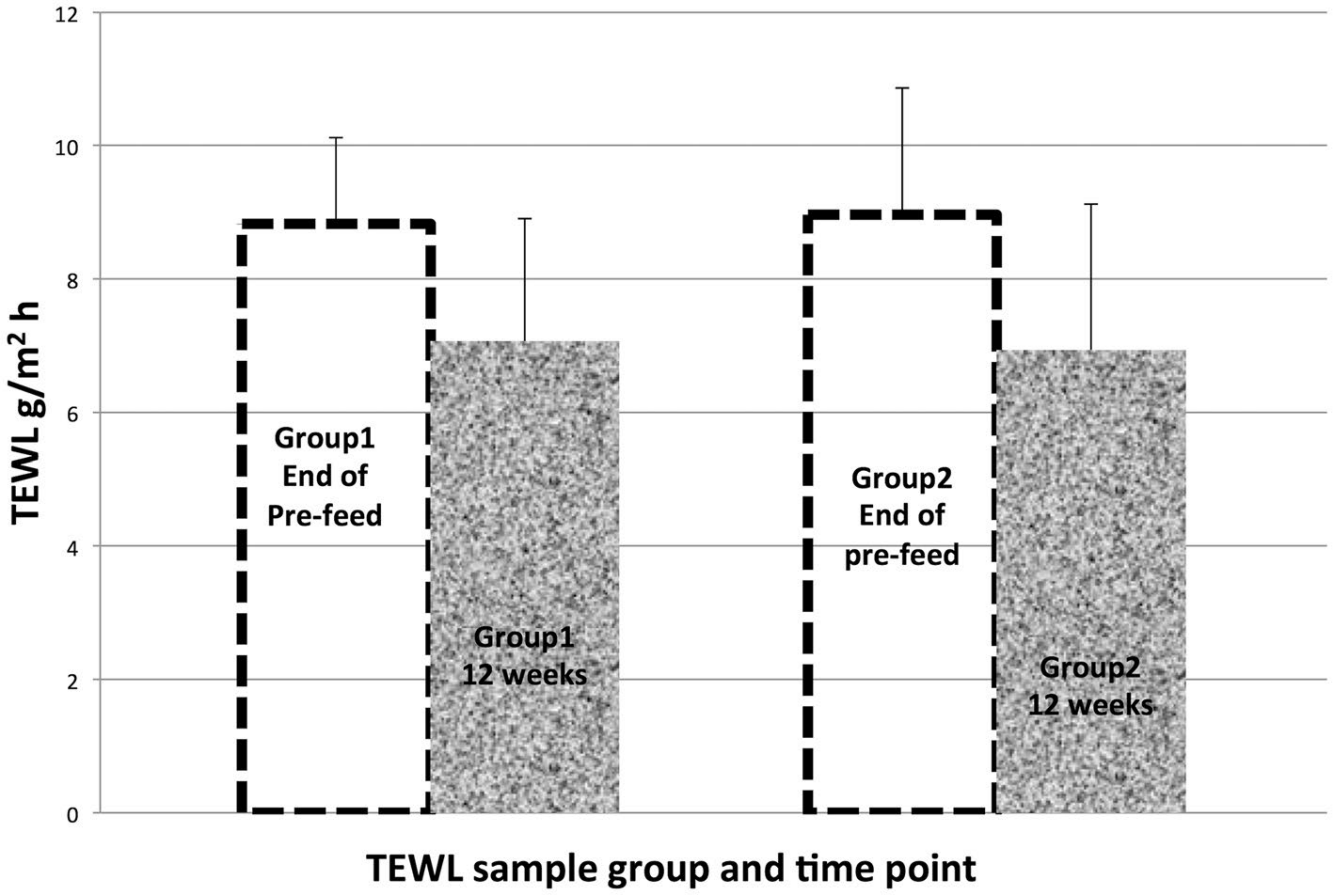
Transepidermal water loss. TEWL observed for the stratum corneum of dogs from Group 1 vs. Group 2 at the end of the 12 weeks pre-feed period and following 12 weeks of differential feeding (Mean ± SEM; *n* = 5)

Although the increases in linoleic acid and ceramide content were significant in the SC of dogs fed for 12 weeks with the linoleic acid-enriched diet, we were not able to detect an associated differential change in barrier properties between the two groups and there was no sign of beneficial effects on the skin of these normal dogs. No difference in the variation of the epidermal water loss was observed in both dogs groups (Fig. 8), and this suggests a close relationship with the lack of change of the protein-bound ceramide content of the SC.

## Discussion

We have investigated the role of dietary LA in skin homeostasis by comparing lipid metabolism when feeding an adequate versus a LA supplemented diet to healthy adult Labradors. Feeding the dogs with the enriched diet for 12 weeks resulted in an increase in linoleic acid within the SC but the supplemented regime did not change the proportion of linoleic acid present in acylacids, which remained tenfold higher than that found as free acid. The latter observation raises a question regarding the mechanisms of biosynthesis leading to the constant proportion of linoleic acid in acylacids. Nutgeren et al. [21] followed the time-dependent incorporation in acylglucosylceramides, acylceramides and acylacids of radioactive linoleic acid topically applied to the skin of fatty acid-deficient rats. Their data showed that exogenous linoleic acid is first found in epidermal acylglucosylceramides, then in acylceramides and finally in acylacids. Our results show that orally fed and topically applied linoleic acid are indeed similarly metabolized. Such a time dependence suggests that acylation by linoleic acid first occurs in the epidermis for molecular species of glucosylceramides containing ω-OH VLCFA. Although the pathway of biosynthesis of the latter molecular species is still to be fully characterized in the epidermis, Takagi et al. [28] showed that they can be formed by omega hydroxylation of the glucosylceramides with VLCFA that are present in a fraction of epidermal glucosylceramides [10]. Acylglucosylceramides are processed via two different pathways. They can be hydrolyzed to acylceramides by a beta-glucocerebrosidase, an enzyme that is very active in the stratum corneum [27], before being cleaved into long-chain bases and acylacids by an acid ceramidase in the upper layers of epidermis, as suggested by Houben et al. [12]. In the second pathway, they are coupled to the cornified envelope through transesterification by a glutaminase, thus releasing the linoleic acid, before a glucocerebrosidase cleaves the glucose and a ceramidase releases the sphingoid base [30]. This second pathway is likely to be effective in healthy epidermis where there is a sufficient content in acylglucosylceramides to ensure a proper formation of the cornified envelope, and an additional supply of LA should lead to the storage of linoleic acid in the form of acylacids, which seems to be the prevailing storage form in the present study.

Glucosylceramides are found in epidermis, but not in healthy SC that contains only ceramides as sphingolipids as shown by our previous studies [23] and others [9–11]. A simultaneous increase of acylglucosylceramides and acylceramides induced by the LA-enriched diet would be an interesting hypothesis. However, that possibility can only be assessed by analyzing samples of the total epidermis, which was excluded owing to the design of the current study.

The preferential storage of linoleic acid as an ester to ω-OH VLCFA in the stratum corneum implies that such fatty acids are available. Since the amount of free ω-OH VLCFA present in the free fatty acid fraction (Fig. 5) is very low, this suggests that the biosynthesis of these fatty acids is stimulated by the increase in the concentration of linoleic acid upon feeding with a linoleic acid-enriched diet. What is clear is that the increased amount of linoleic acid is also stored as an ester. This might be rationalized by the fact that the skin lipoxygenases, such as 12R-LOX, responsible for producing the oxygenated derivatives of linoleic acid required to ensure a proper water barrier, are active only when linoleic acid is in an esterified form [26].

The observed increase of total SC ceramides after the supplementary feeding of linoleic acid for three months is consistent with the reported finding of increased ceramides in the skeletal muscles of humans fed three months with linoleic acid at 4 g per day [29]. Linoleic acid is a potent ligand of the PPARSα [8] that are known to be progressively upregulated during keratinocyte differentiation [24]. Selective activation of PPARSα was found to induce a significant increase of all ceramides, including acylceramides [25], without any effect on the free cholesterol and free fatty acids contents. This is consistent with our present findings. It is interesting that in the same study β-glucocerebrosidase was shown to be strongly upregulated along with serine palmitoyltransferase, the enzyme that catalyses the first step in the biosynthesis of the long-chain bases of ceramides. It is noteworthy that the protein-bound ceramide content that is necessary for the formation of an efficient cornified envelope was not modified by the linoleic acid-enriched diet, and it could be expected that, since this study dealt with healthy dogs, the cutaneous barrier function was optimal and there should not have been any change in the TEWL of the dogs used in this study. The lack of detectable differences in water loss between the groups is presumably the result of an adequate balanced diet, despite differential LA intake, being fed to these dogs. An alternative methodology is to apply a perturbation to the skin, for example, chemical lipid disruption, and subsequently measure rate of recovery [17]. The method could be useful for understanding the underlying reservoir of lipids that can restore skin homeostasis, and may therefore, have been more elucidatory in this instance. It seems clear that in canine or human skin pathologies, the homeostasis of SC is a critical determinant of disease severity and duration [16, 30]. The present study could provide useful insights to improve our understanding of epidermal linoleic acid metabolism and ultimately how we optimize essential fatty acid intake for health and disease. In humans, the current consumption of linoleic acid in the U.S. is 16 g per day by average men and 12.6 g per day by women [2]. These intake levels recorded for humans is reported as between 5 and 6% of metabolisable energy, and this would compare to between 4 and 7 percent for the range of diets commonly fed to dogs [15]. As there are no published similar studies on the linoleic acid content of human SC, the extrapolation of the present results to humans is difficult. However, the fact that a high linoleic acid content in the diet increases the ceramides in canine SC could be potentially interesting in the treatment of atopic dermatitis in humans which is known to be associated with a decreased SC ceramide content [7, 13].

In conclusion, it is likely that in canine or human skin disease, the homeostasis of SC is an important factor to be taken into consideration. The present study brings new insights to the role of linoleic acid intake in lipid metabolism in the skin. Increasing dietary intake of linoleic acid increased LA content of the SC accounted for principally by its conversion into the acylacid form. Whereas the protein-bound ceramides remained stable, there was also evidence for an increase in the overall ceramide content of the SC that did not induce a measurable change in barrier function.

## Abbreviations

ω-OH VLCFA: Omega-hydroxy very long chain fatty acids
TEWL: Transepidermal water loss
LA: Linoleic acid
PPARSα: Peroxisome proliferator-activated receptors alpha

## References

[1] Ahlstrøm Ø, Krogdahl A, Vhile SG, Skrede A (2004). Fatty acid composition in commercial dog foods. J Nutr.

[2] Barnard ND (2010). Trends in food availability, 1909–2007. Am J Clin Nutr.

[3] Bowser PA, Nugteren DH, White RJ, Houtsmuller UM, Prottey C (1985) Identification, isolation and characterization of epidermal lipids containing linoleic acid. Biochim Biophys Acta 834: 419 – 28. 10.1016/0005-2760(85)90016-5 10.1016/0005-2760(85)90016-5 3995076

[4] Butovich IA, Wojtowicz JC, Molai M (2009). Very long chain wax esters and (O-acyl)-omega-hydroxy fatty acids of meibum. J Lipid Res.

[5] Cosgrove MC, Franco OH, Granger SP, Murray PG, Mayes AE (2007). Dietary nutrient intakes and skin-aging appearance among middle-aged American women. Am J Clin Nutr.

[6] Cunnane S, Anderson M (1997). Pure linoleate deficiency in the rat: influence on growth, accumulation of n-6 polyunsaturates, and (1-14C) linoleate oxidation. J Lipid Res.

[7] Di Nardo A, Wertz P, Gianneti A, Seidenari S (1998). Ceramide and cholesterol composition of the skin of patients with atopic dermatitis. Acta Derm Venereol.

[8] Forman BM, Chen J, Evans RM (1997). Hypolipidemic drugs, polyunsaturated fatty acids, and eicosanoids are ligands for peroxisome proliferator-activated receptors α and δ. Proc Natl Acad Sci USA.

[9] Gray GM, White RJ (1978). Glycosphingolipids and ceramides in human and pig epidermis. J Invest Dermatol.

[10] Hamanaka S, Hara M, Nishio N, Otsuka F, Suzuki A, Uchida Y (2002) Human epidermal glucosylceramides are major precursors of stratum corneum ceramides. J Invest Dermatol 119: 416 – 23. 10.1046/j.1523-1747.2002.01836.x 10.1046/j.1523-1747.2002.01836.x 12190865

[11] Holleran WM, Takagi Y, Menon GK, Legler G, Feingold KR, Elias PM (1993). Processing of epidermal glucosylceramides is required for optimal mammalian cutaneous permeability barrier function. J Clin Invest.

[12] Houben E, Uchida Y, Nieuwenhuizen WF (2007). Kinetic characteristics of acidic and alkaline ceramidase in human epidermis. Skin Pharmacol Physiol.

[13] Imokawa G, Abe A, Jin K, Higaki Y, Kawashima M, Hidano A (1991). Decreased levels of ceramides in stratum corneum of atopic dermatitis: an etiologic factor in atopic dry skin?. J Invest Dermatol.

[14] Imokawa G, Yada Y, Higuchi K, Okuda M, Ohashi Y, Kawamata A (1994). Pseudo-acylceramide with linoleic acid produces selective recovery of diminished cutaneous barrier function in essential fatty acid-deficient rats and has an inhibitory effect on epidermal hyperplasia. J Clin Invest.

[15] Jandacek RJ (2017). Linoleic acid: a nutritional quandary. Healthcare (Basel).

[16] Marsella R (2013) Fixing the skin barrier: past, present and future—man and dog compared. Vet Dermatol 24: 73 – 6. 10.1111/j.1365-3164.2012.01073.x 10.1111/j.1365-3164.2012.01073.x 23331682

[17] Meguro S, Arai Y, Masukawa Y, Uie K, Tokimitsu I (2000). Relationship between covalently bound ceramides and transepidermal water loss (TEWL). Arch Dermatol Res.

[18] Melton JL, Wertz PW, Swartzendruber DC, Downing DT (1997). Effects of essential fatty acid deficiency on epidermal O-acylsphingolipids and transepidermal water loss in young pigs. Biochim Biophys Acta.

[19] National Research Council (2006). Nutrient requirements of dogs and cats.

[20] Nicollier M, Massengo T, Rémy-Martin JP, Laurent R, Adessi GL (1986). Free fatty acids and fatty acids of triacylglycerols in normal and hyperkeratotic human stratum corneum. J Invest Dermatol.

[21] Nugteren DH, Christ-Hazelhof E, Van der Beek A, Houtsmuller UMT (1985). Metabolism of linoleic acid and other essential fatty acids in the epidermis of the rat. Biochim Biophys Acta.

[22] Popa I, Thuy LH, Colsch B, Pin D, Gatto H, Haftek M, Portoukalian J (2010). Analysis of free and protein-bound ceramides by tape stripping of stratum corneum from dogs. Arch Dermatol Res.

[23] Popa I, Remoue N, Osta B, Pin D, Gatto H, Haftek M, Portoukalian J (2012). The lipid alterations in the stratum corneum of dogs with atopic dermatitis are alleviated by topical application of a sphingolipid-containing emulsion. Clin Exp Dermatol.

[24] Rivier M, Safonova I, Lebrun P, Griffiths CE, Ailhaud G, Michel S (1998). Differential expression of peroxisome proliferator-activated receptor subtypes during the course of keratinocyte differentiation. J Invest Dermatol.

[25] Rivier M, Castiel I, Safonova I, Ailhaud G, Michel S (2000). Peroxisome proliferator-activated receptor-α enhances lipid metabolism in a skin equivalent model. J Invest Dermatol.

[26] Siebert M, Krieg P, Lehman WD, Marks F, Fürstenberger G (2001). Enzymic characterization of epidermis-derived 12-lipoxygenase isoenzymes. Biochem J.

[27] Takagi Y, Kriehuber E, Imokawa G, Elias PM, Holleran WM (1999). β-glucocerebrosidase activity in mammalian stratum corneum. J Lipid Res.

[28] Takagi Y, Nakagawa H, Matsuo N, Nomura T, Takizawa M, Imokawa G (2004). Biosynthesis of acylceramide in murine epidermis: characterization by inhibition of glucosylation and deglucosylation, and by substrate specificity. J Invest Dermatol.

[29] Thrush AB, Chabowski A, Heigenhauser GJ, McBride BW, Or-Rashid M, Dyck DJ (2007). Conjugated linoleic acid increases skeletal muscle ceramide content and decreases insulin sensitivity in overweight, non-diabetic humans. Appl Physiol Nutr Metab.

[30] Uchida Y, Holleran WM (2008). Omega-O-acylceramide, a lipid essential for mammalian survival. J Dermatol Sci.

[31] Van Smeden J, Bouwstra JA (2016). Stratum corneum lipids: their role for the skin barrier function in healthy subjects and atopic dermatitis patients. Curr Probl Dermatol.

[32] Wertz PW, Downing DT (1983). Acylglucosylceramides of pig epidermis: structure determination. J Lipid Res.

